# Advancements in diagnosis, preventive care, and future directions in the holistic management of pediatric asthma

**DOI:** 10.3389/fped.2026.1793567

**Published:** 2026-05-29

**Authors:** Wenwen Guo, Kangjian Li

**Affiliations:** 1 Department of Pre-school Education and Early Childhood Care Management, Jining Polytechnic, Jining, China; 2 Pediatric Department, Guangdong Provincial Hospital of Chinese Medicine, Guangzhou, China

**Keywords:** airway inflammation, asthma prevention, asthma treatment, healthcare system, pediatric asthma

## Abstract

Pediatric asthma is a highly prevalent chronic respiratory disorder characterized by airway inflammation, hyperreactivity, and variable airflow limitation, resulting in significant global health and socioeconomic burdens. Advances in diagnosis, including the measurement of fractional exhaled nitric oxide, assessment of blood eosinophils, and artificial intelligence-assisted tools, have improved early detection and disease stratification. Optimal management combines planned daily care, which includes systematic symptom monitoring, adherence to prescribed medications, frequent physical activity, and the promotion of adequate sleep. Targeted preventative methods, including allergy avoidance, viral infection prophylaxis, air quality improvement, and lifestyle optimization, reduce exacerbation risk and promote long-term control. Multiple variables influence pediatric asthma outcomes, including genetic predisposition, environmental exposures, and social determinants, emphasizing the significance of coordinated care among caregivers, schools, and multidisciplinary healthcare teams. Stepwise pharmacological and non-pharmacological therapies provide tailored treatment options for severe or refractory conditions. Persistent barriers to healthcare access, disparities in disease burden, and issues with treatment adherence underline the need for creative solutions. Emerging approaches, such as digital self-management tools, precision-based prevention strategies, and community-level interventions, present prospects to improve disease control. Effective asthma care goes beyond pharmacological treatment, focusing on early intervention, personalized support, and equitable access to improve children's long-term respiratory health and quality of life.

## Graphical Abstract

**Figure:**
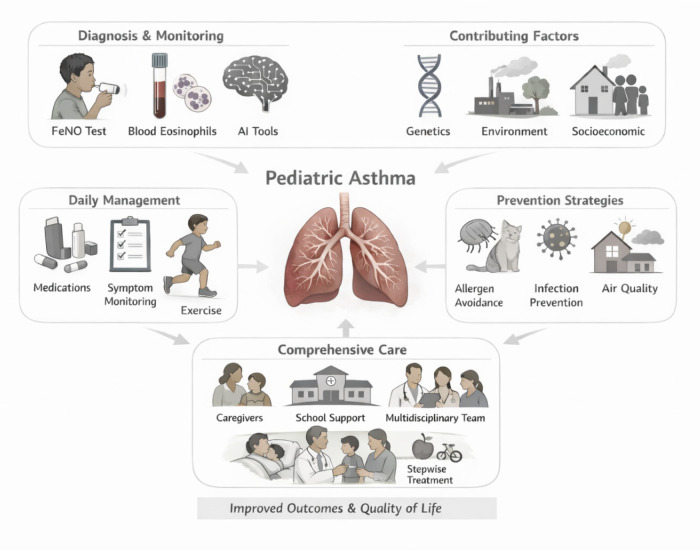
This graphical abstract presents the core components of pediatric asthma care: diagnosis/monitoring, risk factors, daily management, prevention strategies, and comprehensive care (including caregivers, school support, and multidisciplinary teams). Together, these integrated approaches are designed to enhance outcomes and quality of life for children with asthma.

## 1 Introduction

Pediatric asthma is a highly common chronic respiratory condition among children globally, putting a considerable strain on global public health, individual well-being, and healthcare systems (1). This respiratory disorder is characterized by persistent airway inflammation, hyperreactivity, and variable airflow limitation. The disorder presents with recurrent symptoms such as wheezing, coughing, shortness of breath, and chest tightness, which may impair daily activities and long-term lung health if inadequately managed (2). Beyond the physical toll, uncontrolled asthma impairs children's quality of life, hinders academic achievement, and imposes emotional stress on both patients and their caregivers, emphasizing the urgent need for comprehensive, evidence-based care options (3).

The paradigm of pediatric asthma management is changing, driven by a greater understanding of the disease's heterogeneity and the promise of precision medicine (4). Traditional stepwise therapy, while helpful for many, fails to account for the multiple inflammatory endotypes driving asthma, including T2-high asthma inflammation, which is characterized by higher levels of cytokines such as IgE, *IL-5, IL-4, and IL-13* (5). This realization has accelerated the development of biologic treatments, which are monoclonal antibodies that target specific mediators of the inflammatory cascade. Biologic therapies have been available to adults for nearly two decades, with increasing evidence and approvals in pediatric populations in recent years (6). Research on their efficacy, safety, and optimal use in younger children has increased dramatically in recent years, bringing new hope for children with severe, uncontrolled asthma (4). By directly addressing pathogenic pathways, biologics try to avoid exacerbations, improve symptom control, and minimize reliance on systemic corticosteroids, addressing unmet requirements in severe childhood asthma therapy (7). Against this context, early detection, proactive monitoring, and preventative interventions emerge as critical components of effective pediatric asthma treatment. Timely diagnosis and treatments not only ease acute symptom load but also reduce the risk of long-term lung function deterioration, which is a key concern in pediatric populations where lung development is ongoing (8). Asthma education for children and their caregivers is also critical, allowing them to spot triggers, stick to drug regimens, and practice daily care routines that maintain control and prevent exacerbations (9). Environmental and behavioral adjustments, such as minimizing exposure to allergens, air pollution, and viral infections, add to pharmacological interventions, establishing a holistic approach to asthma therapy (5).

The purpose of this review is to summarize the most recent advances in the diagnosis and treatment of pediatric asthma, including pathophysiology insights, diagnostic breakthroughs, therapeutic techniques, and future directions. We hope to present a comprehensive overview of current best practices by looking at the various causes of pediatric asthma, developing biomarkers for early detection, and personalized care for unique populations.

## 2 Review methodology

A structured literature search was carried out across major electronic databases, including PubMed, Embase, Web of Science, and the Cochrane Library, to give a complete evaluation of pediatric asthma treatments. The search included papers from 2000 to 2025 that reflected current diagnostic standards and treatment methodologies. The search phrases “pediatric asthma”, “childhood asthma”, “management”, and “precision medicine” were combined with Boolean operators to capture relevant literature. We prioritized original research, systematic reviews, meta-analyses, and clinical guidelines on the causes, diagnosis, treatment, and epidemiology of asthma in children. Inclusion criteria emphasized studies with well-defined pediatric populations, clinically relevant outcomes, and methodological rigor. Exclusion criteria included adult-only research, case reports, and non-English language publications without translations.

Selected studies were evaluated for relevance and methodological quality, with evidence synthesized using a narrative theme approach rather than a quantitative meta-analysis. Guidelines, randomized controlled trials, and high-quality observational studies were weighted based on their design and relevance, with guideline-based evidence taking precedence for clinical recommendations. Conflicting evidence was noted, and major conclusions were summed up to reflect the current state of knowledge. Although this is not a formal systematic review, the methodology promotes transparency, reproducibility, and critical evaluation, allowing for a credible synthesis of existing information in pediatric asthma care.

## 3 Pathophysiology and core mechanism of pediatric asthma

**Figure 1:**
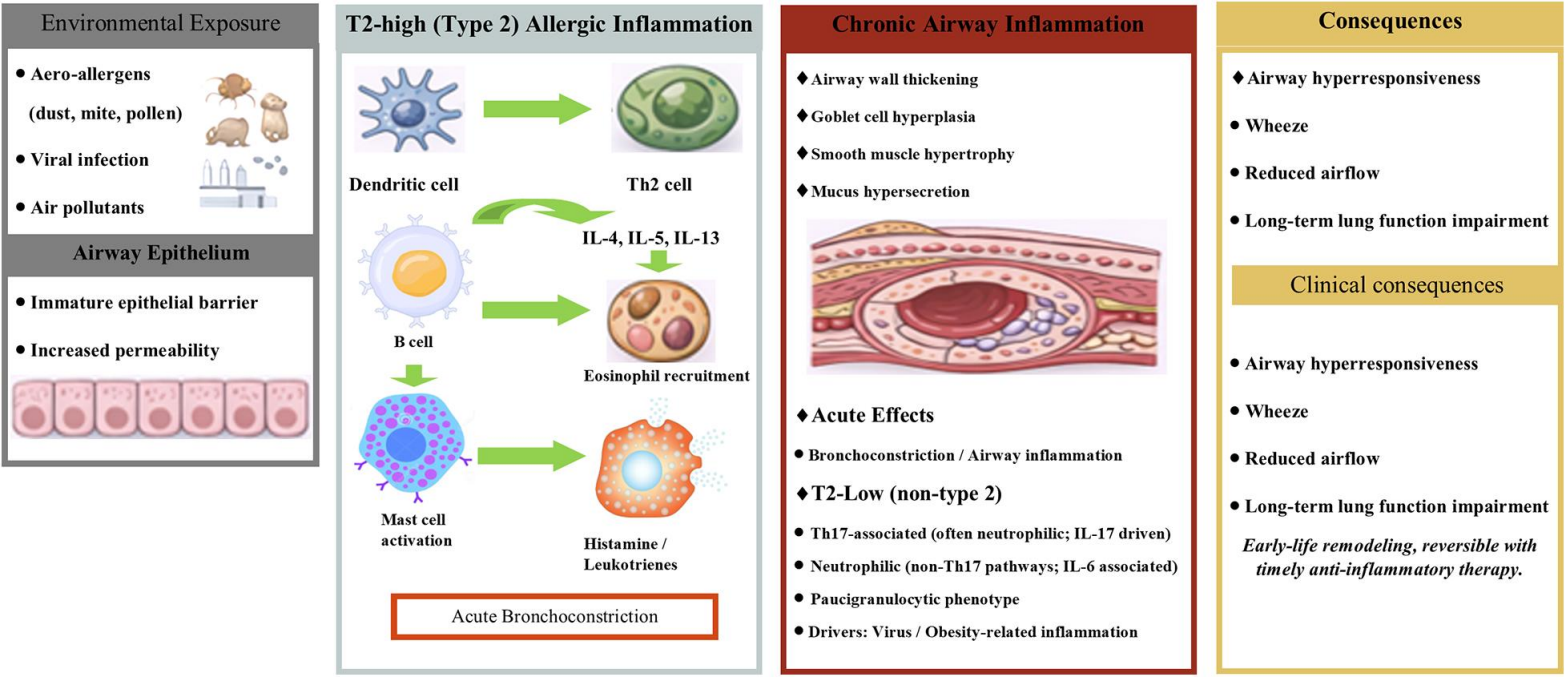
The key physiological and molecular mechanisms underlying the T2-high (allergic) and T2-low (non-type 2) inflammatory phenotypes of chronic airway inflammation in pediatric asthma. Allergens, pollen, and pet dander are examples of environmental stimuli that interact with an immature, porous airway epithelium to activate dendritic cells and promote Th2 differentiation. While IgE-allergen complexes induce mast cell/basophil histamine and leukotriene degranulation, which leads to immediate bronchoconstriction and inflammation, Th2 cells promote B cell IgE production, eosinophil recruitment, and mucus hypersecretion. In contrast, T2-low asthma is an umbrella category that encompasses neutrophilic (both Th17-associated and non-Th17, IL-6-driven) and Paucigranulocytic phenotypes, often induced by viral infections, obesity, and related inflammatory pathways. Both inflammatory pathways contribute to chronic airway changes, including airway hyperreactivity, wheezing, reduced airflow, and long-term deterioration of lung function.

For the successful management of pediatric asthma, it is critical to understand its complex, age-specific pathophysiology, which differs from that of adult asthma due to ongoing lung development, immature immune responses, and unique environmental interactions (10) (Figure 1). Asthma is fundamentally a disease characterized by dysregulated immunology, persistent airway inflammation, and airway hyperreactivity (AHR), with genetic predisposition and environmental triggers driving illness development and progression (4, 11).

The immune system is crucial to pediatric asthma, with a heavy emphasis on allergic inflammation in most children. Children's immune systems, unlike those of adults, are essentially immature, with a leaning toward Th2 (T-helper 2) cell responses–a developmental adaptation that protects against parasite infections but increases sensitivity to allergy sensitization (12). In T2-high asthma, exposure to allergens triggers a cascade of immune events: dendritic cells capture and present allergens to naive T cells, prompting their differentiation into Th2 cells. Th2 cells then secrete *IL-4, IL-5, and IL-13*, where *IL-4* drives B cell class switching to IgE production, *IL-5* promotes eosinophil proliferation and recruitment, and IL-13 induces mucus hypersecretion and airway remodeling (13). Finally, IgE binds to high-affinity receptors on mast cells and basophils, and subsequent allergen exposure cross-links IgE, triggering degranulation and release of pro-inflammatory mediators that lead to acute bronchoconstriction and inflammation (14). While T2-high asthma dominates pediatric cases, non-T2 asthma also occurs, particularly in older children and those with severe disease, characterized by neutrophilic inflammation driven by Th17 cells and cytokines such as IL-6 and IL-17, often linked to viral infections or obesity (15, 16).

Airway remodeling-structural changes to the airway wall-was once thought to be a late feature of asthma, but evidence now confirms it begins in childhood. In pediatric asthma, remodeling includes smooth muscle hypertrophy and hyperplasia, which increase airway narrowing during exacerbations, as well as subepithelial fibrosis, which thickens the basement membrane and reduces airway compliance (17). Mucus gland hyperplasia, which boosts mucus production and airway obstruction, while angiogenesis forms new blood vessels to enhance inflammatory cell recruitment (18). Importantly, remodeling in children is often reversible with early, aggressive-inflammatory therapy, emphasizing the critical role of timely intervention to preserve lung function and prevent long-term structural damage (19).

## 4 Diagnostic advances

Accurate and fast diagnosis of pediatric asthma is crucial for commencing early therapy, minimizing exacerbations, and maintaining lung function (20). However, diagnosing asthma in children is frequently difficult due to age-related restrictions in testing, overlapping symptoms with other respiratory disorders (e.g., viral bronchiolitis, cystic fibrosis), and diverse symptom presentation (21). Over the past decade, diagnostic advances, including improved standard tools and emerging biomarkers, have enhanced our ability to identify asthma in children, even in preschoolers (22).

### 4.1 Current diagnostic tool

A careful clinical history and physical examination are essential for diagnosing pediatric asthma, especially in young children who are unable to conduct objective lung function testing. Key historical features include recurring wheezing, coughing, shortness of breath, and chest tightness (23). In preschoolers, persistent wheezing or wheezing unrelated to colds is more predictive of asthma than occasional wheezing with viral infections. Trigger association-symptoms triggered by allergens, exercise, cold air, smoke, or stress-also support the diagnosis, as does a family history of asthma or atopy and a personal history of atopy (10). Improvement in symptoms following short-acting β2-agonists (SABAs; section 6.1 for pharmacological details) is an important diagnostic clue, confirming reversible airflow restriction (24). On physical examination, acute exacerbations present with wheezing, tachypnea, retractions, and the use of accessory muscles. In contrast, the asymptomatic period often yields a normal examination, although subtle findings such as hyperresonance to percussion or prolonged expiration may be present (25).

Although lung function testing offers objective proof of airflow restriction and reversibility, its applicability varies with age. For children aged 6 years and above, spirometry is the gold standard for assessing lung function indices, such as forced vital capacity (FVC) and forced expiratory volume in one second (FEV_1_) (26). Airflow limitation is indicated by a decreased FEV_1_/FVC ratio (below the lower limit of normal for age and sex). However, the diagnosis of asthma is not based on a single parameter, but rather on the demonstration of variable expiratory airflow limitation in the appropriate clinical context. This variability can be supported by bronchodilator reversibility (e.g., an increase in FEV_1_ of ≥12% and ≥200 mL from baseline following inhalation of SABA), excessive peak expiratory flow variability, and improvement in lung function after a trial of anti-inflammatory treatment (27). Importantly, these objective measures should be interpreted alongside clinical features, as a normal spirometry does not exclude asthma, particularly in children. For children with normal baseline spirometry but suspicious symptoms, bronchoprovocation testing with methacholine or mannitol is utilized. A ≥ 20% fall in FEV_1_ during bronchoprovocation supports airway hyperresponsiveness consistent with asthma (26, 28). Using a peak flow meter is suitable for children aged 6–7 years who can perform consistent blows (29). For children under <6 years, standard spirometry is feasible, but challenging, so alternatives like the forced oscillation technique (FOT) and impulse oscillometer (IOS) are used (30). FOT measures airway resistance and reactance using pressure oscillations, requiring minimal cooperation, while IOS uses impulse signals to assess central and peripheral airway function; both are useful for detecting small airway disease common in pediatric asthma (31).

Pediatric asthma must be distinguished from other respiratory conditions with similar symptoms to avoid misdiagnosis (32). Viral bronchiolitis is common in infants <1 year, with symptoms resolving within 1–2 weeks. However, recurrent bronchiolitis may predict asthma, but is not diagnostic. Cystic fibrosis (CF) presents with chronic cough, wheezing, and recurrent infections, diagnosed via sweat chloride test or CFTR gene testing (33, 34). Primary ciliary dyskinesia (PCD) is characterized by chronic sinusitis, otitis media, and bronchiectasis, diagnosed via nasal nitric oxide (nNO) testing and ciliary motility analysis. Aspiration, due to gastroesophageal reflux (GERD) or swallowing dysfunction, presents with recurrent pneumonia, coughing during feeding, or nocturnal cough (35). Vocal cord dysfunction (VCD) causes “wheezing” from vocal cord narrowing, worsening with stress or exercise, and is diagnosed via laryngoscopy. A thorough differential diagnosis ensures that children receive appropriate targeted care (36).

### 4.2 Emerging biomarkers and diagnostic tools

Biomarkers provide objective measures of inflammation, endotype, and disease activity, addressing limitations of clinical history and lung function testing, while emerging technologies are particularly valuable for young children and those with atypical presentations (37, 38) (Table 1). Fractional exhaled nitric oxide (FeNO) is a non-invasive biomarker of T2-high asthma (Eosinophilic) inflammation: nitric oxide is produced by airway epithelial cells in response to IL-4 and IL-13, so elevated FeNO indicates ongoing eosinophilic inflammation (39). FeNO cut-offs are age-dependent in pediatric populations and not restricted to a single age threshold. Its non-invasive nature and minimal cooperation requirement make it a valuable tool in pediatric practices (40).

**Table 1:** Diagnostic biomarkers of pediatric asthma and its response to various treatments.

Biomarkers	Phenotype	Treatment response	Relation with other markers
Blood eosinophils	Type 2 Asthma	Helps predict response to ant-IL-5 therapy	Positively linked with FeNO and IL-5
FeNO	Type 2 Asthma		Shows correlation with eosinophils and IL-13
Serum IgE	Allergic Asthma	Predicts response to anti-IgE therapy	Shows correlation with eosinophils
IL-5	Type 2 Asthma	Target of anti-IL-5 therapies	Correlated with blood eosinophilic counts
IL-13	Type 2 Asthma	Targeted by anti-IL4 R*α*	Associated with FeNO
TSLP	Early-stage inflammation	Target of anti-TSLP therapy	Affects both eosinophilic and neutrophilic pathways
Blood neutrophils	Non-type 2 asthma	Limited effect from current biologics	Linked with IL-8 and airway remodeling
IL-33	Innate immunity	Investigated as a biological target	Interacts with TSLP in inflammatory pathways
IL-8	Neutrophilic inflammation	Not targeted by available biologics	Correlates with neutrophilic phenotype

With a pediatric reference range of less than 400 cells/μL in the majority of populations, the peripheral blood eosinophil count is an easily accessible and clinically valuable biomarker of T2-high asthma inflammation in children. This cutoff, however, varies by age, laboratory, and clinical situation and is not always conclusive. In children who are not receiving treatment, counts greater than 400 cells/uL are linked to eosinophilic asthma and an elevated risk of exacerbations (41). Acute infections, allergic reactions, non-asthmatic inflammatory diseases, and recent use of systemic or inhaled corticosteroids (which lower circulating eosinophils) are among the confounding factors that can affect dynamic eosinophil levels (42). Eosinophil counts support the clinical suspicion of T2-high asthma in children with recurrent wheeze (when interpreted with FeNO and clinical history) and guide biologic therapy selection. However, eosinophil counts can be elevated by allergies, parasitic infections, or other inflammatory conditions. Sputum eosinophil count is the gold standard for assessing airway eosinophilic inflammation, but its utility in children is limited by difficulty in obtaining sputum samples. For cooperative children >6 years, sputum eosinophils >3% indicate T2-high asthma and predict response to ICS (43, 44).

Emerging technologies are expanding the diagnostic toolkit for pediatric asthma. Exhaled breathomics analyzes VOCs in exhaled breath to identify asthma-specific patterns, distinguishing asthma from other respiratory conditions and predicting exacerbations (45). Ongoing research is confirming the clinical efficacy of this non-invasive technique, which is appropriate for young infants (46). Rapid, on-site biomarker testing in clinics or schools is made possible by point-of-care (POC) testing, portable FeNO devices, and POC eosinophil analyzers, increasing access to diagnostic tools in underserved locations (40). Aligned with the Healthcare 5.0 framework, which prioritizes human-centered care, equity, and clinical integration, artificial intelligence (AI) and machine learning algorithms are transforming pediatric asthma diagnosis by combining clinical history, physical examination, lung function, and biomarker data into interpretable, clinician-friendly models (38). These human-centered AI tools improve diagnostic timeliness and accuracy, especially for preschoolers with atypical presentations, and are intended to promote health equity by standardizing diagnostic workflows in underserved clinical settings with limited access to pediatric asthma specialists (47). Healthcare 5.0-aligned models improve translational relevance and trust among physicians and caregivers by incorporating AI into everyday diagnostic care. However, as recent research has shown, defining AI simply in terms of accuracy risks disregarding key intersectionality, health disparities, and ethical implications (48). AI models trained on homogeneous datasets (e.g., underrepresenting racial/ethnic minorities, low-income children, or rural populations) have the potential to perpetuate or amplify existing disparities. Ethical concerns include ensuring informed caregiver consent for pediatric data use, protecting sensitive health information, and maintaining transparency in AI-driven diagnostic decisions (49). Responsible AI adoption in pediatric asthma demands prioritizing diverse, representative training data, undertaking routine bias audits, and engaging underrepresented communities to ensure technologies fit their unique needs, thereby improving equity alongside accuracy (38).

## 5 Daily care, health maintenance, and prevention

Pediatric asthma management goes beyond medicines. Daily care, health maintenance, and prevention are essential for managing symptoms, minimizing exacerbations, and maintaining lung function. This “holistic” approach enables children and caregivers to actively participate in disease management, boosting independence and enhancing quality of life (20).

### 5.1 Organized daily care schedules

#### 5.1.1 Lung function monitoring and symptoms

Effective daily asthma management is built on routine, structured monitoring because it detects early indicators of deteriorating control and enables proactive action before symptoms develop (4). Peak flow monitoring (PFM) is an easy-to-use, practical instrument for children aged ≥ 5. Tracking airflow stability involves measuring PEF in the morning and evening, logging numbers in a log or app, and comparing them to a personal best (50). Even in the absence of symptoms, a decline in PEF greater than 15% from personal best suggests early deterioration, which prompts caretakers to modify rescue medicine use or get in touch with a provider. Symptom diaries are very helpful for younger kids or those who cannot use a peak flow meter. By keeping note of coughing, wheezing, shortness of breath, SABA usage, and activity limitations during the day and at night, caregivers may create a clear picture of disease activity over time (51). By creating visual reports for providers, indicating troubling trends, and sending reminders to collect data, digital solutions like asthma management apps make monitoring easier. The number of SABA doses taken each week, the frequency of nocturnal awakenings, and the number of school/activity days missed are important metrics to emphasize since they have a high correlation with overall control and exacerbation risk (52). Digital health tools and AI-based monitoring systems, developed within the Healthcare 5.0 framework of human-centeredness and personalization, show promise for automating data collection, generating individualized asthma exacerbation alarms, and providing tailored self-management guidance to caregivers and children (53, 54). This Healthcare 5.0 alignment guarantees that AI tools prioritize clinical integration by embedding into routine symptom/PEF monitoring procedures rather than acting as standalone technical solutions (55). However, their applications are not without limitations. Many digital technologies and AI models have received insufficient validation in diverse pediatric groups, raising concerns about accuracy and reliability, and fair access to these tools remains a crucial issue. Healthcare 5.0 is a barrier for low-income and rural families (56).

#### 5.1.2 Optimizing medication adherence

The most important factor in controlling asthma is medication adherence, although non-adherence is still a major problem for children of all ages. Routines should smoothly incorporate medicine administration into everyday living to alleviate this and lessen reliance on memory. To help toddlers form habits, associate dosages with regular cues like mealtimes or bedtimes (57). Involving school-age children in the process by giving them a choice between a colorful inhaler mask and a “medication timer” watch, which encourages ownership. While portable inhalers with built-in sensors offer feedback on use and send reminders, adolescents benefit from discrete, practical solutions like once-daily ICS/LABA combinations that lessen the load (57, 58). Directly addressing obstacles is crucial when it comes to financial worries, looking into generic drugs, or patient support programs. When people are afraid of adverse effects, reassure them that using prescriptions correctly reduces risks. Additionally, caregivers can set an example of adherence by regularly giving prescription drugs as directed, which helps kids understand how important they are (58).

#### 5.1.3 Physical activity integration with exercise

Regular physical activity is essential for children with asthma because it strengthens respiratory muscles, improves lung function, and promotes emotional well-being. Exercise-induced bronchoconstriction (EIB) frequently discourages participation. A proactive exercise plan involves pre-exercise preventive measures, such as inhaling SABA 15–20 min before activity, or employing a daily leukotriene modulator for chronic EIB. Maintenance controller therapy with ICS or ICS/LABA is an important part of EIB management. It reduces baseline airway inflammation and thus the susceptibility to exercise-induced symptoms (59). Alongside regular controller therapy with ICS or ICS/LABA to reduce underlying airway inflammation gradual warm-ups, such as walking, stretching, or mild play, and cool-down intervals, help adapt airways to higher ventilation, lowering EIB risk (60). In cold conditions, youngsters might wear a scarf or mask to warm their breath. During high pollen or pollution days, choose indoor activities such as swimming or yoga. Coaches, teachers, and peers should be trained to promote inclusive participation by providing breaks, medicine access, and minimizing the urge to “push through” symptoms. Celebrating exercise accomplishments, such as finishing a soccer game without rescue medication, communicates that asthma does not have to limit physical activity (1, 60).

#### 5.1.4 Managing nocturnal symptoms through sleep hygiene

Coughing, wheezing, and nightly awakenings are all signs of poor asthma control and can interfere with undisturbed sleep, which is necessary for growth and immunological function. A sleep-focused program should begin with prescribed controller drugs in the evening, as they lower nighttime airway inflammation (61). To reduce triggers, improve the sleep environment by removing dust-collecting things like stuffed animals and carpets, using allergen-proof mattress and pillow coverings, and keeping humidity between 40% and 60% to prevent airways from drying out (62). Establishing consistent pre-bedtime behaviors, such as avoiding heavy meals, or strenuous activity two to three hours before bed and including soothing activities like reading or mild stretching, can help reduce stress and physical stimulation that may exacerbate symptoms. For children who awake with asthma symptoms, keeping a rescue inhaler within easy reach and noting nocturnal symptoms in a diary may assist healthcare providers in changing treatment methods to promote uninterrupted sleep (61).

### 5.2 Targeted preventative interventions

#### 5.2.1 Allergen-specific prophylaxis

Allergen exposure is a significant cause of childhood asthma. Targeted prevention focuses on avoiding interaction with identified allergens in the home, school, and community. Dust mites are the most common indoor allergen, and key interventions to limit their presence include washing bedding in hot water (60°C) weekly, using allergen-proof covers on mattresses and pillows, vacuuming with HEPA filters, and reducing carpet and upholstery in bedrooms, as dust mites thrive in warm, humid environments (62). Keeping pets out of bedrooms and utilizing HEPA air purifiers in living spaces helps children with pet allergies limit dander exposure. Mold prevention entails repairing leaks quickly, washing moldy surfaces with vinegar or hydrogen peroxide, and using dehumidifiers in wet locations like basements and bathrooms (63). Monitor pollen counts using apps or local weather forecasts, and limit outside activities during peak times. Closing windows and utilizing air conditioning during heavy pollen seasons helps limit indoor exposure (62).

#### 5.2.2 Prevention of viral infections

Respiratory virus Infections such as rhinovirus, RSV, and influenza, are the major causes of asthma exacerbations, making prevention a critical component of asthma treatment. Annual influenza vaccines plus RSV prophylaxis (palivizumab) for high-risk newborns greatly lowers the infection risk (64). Hand hygiene is a basic but effective step. Teach children to wash their hands for at least 20 s with soap and water, or to use an alcohol-based hand sanitizer if soap is not available, before eating, after using the restroom, and after coming into contact with someone who is ill. Avoiding direct contact with sick people, especially during peak cold and flu seasons, helps to decrease exposure. Staying home from school when unwell protects children with asthma from spreading illness and extra infections that could worsen asthma (65). Caregivers should also be educated on recognizing early indicators of viral illness, such as a runny nose or low-grade fever, and initiating preventive measures like boosting fluid intake and using saline nasal drops to prevent progression to asthma (66).

#### 5.2.3 Environmental pollution remediation

Indoor and outdoor air pollution exacerbates asthma inflammation, which is critical for lung health (62). Monitor air quality indexes (AQI) and limit outside activities when the AQI exceeds 150 (which is considered harmful). On high-pollution days, keep windows closed, use HEPA-filtered air purifiers indoors, and avoid intense activities that raise respiratory demand. Reducing indoor pollution focuses on removing sources, such as prohibiting smoking in homes and cars, because secondhand and thirdhand smoke are significant airway irritants. Avoid scented candles, air fresheners, and harsh cleaning chemicals; instead, choose fragrance-free, non-toxic alternatives (67). Ensure sufficient ventilation while cooking by utilizing range hoods to remove smoke and odors. To limit exposure to PM2.5 and VOCs in children who live near highways or industrial regions, utilize air purifiers that include both HEPA and activated carbon. Planting air-purifying plants in homes, such as spurge plants or peace lilies, may help reduce indoor pollutants, but they should not replace HEPA filters as the major mitigation measure (62, 67).

#### 5.2.4 Life-based prevention

Healthy lifestyle behaviors boost immune function, reduce inflammation, and lower asthma risk and severity. Nutrition has an important role. A diet high in fruits and vegetables, whole grains, and omega-3 fatty acids contains antioxidants and anti-inflammatory chemicals that promote airway health (68). Limiting processed meals, added sugars, and trans fats, all of which promote inflammation, can help you maintain good asthma control. Many children with asthma are deficient in vitamin D, therefore supplementation lessens the incidence of exacerbations (69). Weight loss is critical for obese children because it improves lung function, medication response, and symptom control. This is accomplished by a balanced diet and regular physical activity tailored to the child's asthma requirements (70). Chronic stress affects the sympathetic nervous system, which worsens airway hyperreactivity. Teaching age-appropriate stress reduction skills, such as deep breathing, mindfulness, art, or sports, can help youngsters deal with stressors like schoolwork or family strife (71). Ensuring proper sleep is also vital: school-age children need 9–12 h of sleep per day, while adolescents require 8–10 h, since sufficient rest promotes immune function and decreases inflammation, further decreasing (72).

### 5.3 Collaboration between caregivers and communities to ensure long-term care

#### 5.3.1 Caregivers' education and empowerment

Caregivers are the foundation of pediatric asthma care, so educating and empowering them is crucial for long-term success. Education should be comprehensive, addressing illness fundamentals such as inflammation, triggers, symptom development, and medication delivery. Caregivers must also learn how to spot early warning symptoms of deteriorating asthma vs. severe exacerbations, and when to take rescue medication or contact emergency services (73). The asthma action plan (AAP) is an important tool. Caregivers should be given a written, color-coded AAP and trained to utilize it to change care depending on symptoms or peak expiratory flow (PEF) measurements. Follow-up education sessions at each provider visit reinforce essential messaging while also addressing new difficulties, such as school transfers or newly recognized triggers. Support groups for caregivers, whether in-person or online, provide a forum for sharing experiences, exchanging tips, and reducing feelings of isolation. Caregivers should also be encouraged to advocate for their child by interacting with schools, childcare providers, and healthcare teams to provide consistent, coordinated care in all settings (74).

#### 5.3.2 School and childcare collaboration

Asthma management must extend to schools and daycare facilities. Share the asthma action plan with staff, including medication lists, trigger avoidance tactics, and emergency protocols. Staff members should be taught to spot signs, deliver rescue medication, and respond to exacerbations (75). Young kids benefit from clearly labeled medications and a dedicated “safe space” for symptom management, whereas older children should be taught to self-manage by carrying their inhaler and monitoring symptoms. Asthma-friendly school regulations, such as clean, well-ventilated classrooms and access to water and rest periods, further enhance children's health (76).

#### 5.3.3 Coordination across healthcare teams

Asthma care requires a multidisciplinary team that includes pediatricians, pulmonologists, nurses, allergists, nutritionists, and mental health experts. Routine management and control monitoring are overseen by primary care professionals, while severe instances are referred to specialists (52). Nurses provide instruction and follow-up, nutritionists advise on nutrition and weight control, and mental health professionals treat anxiety or stress. Telehealth can improve coordination for rural families. Regular team meetings for children with severe asthma ensure that all elements of care are addressed thoroughly (77).

#### 5.3.4 Community and policy cooperation

Sustained asthma management involves both community and policy levels. Community programs can provide resources to impoverished families, such as HEPA filters, allergen-free mattresses, and pharmaceutical assistance. Libraries, community institutions, and houses of religion offer educational workshops that make information accessible (78). Interventions from policymakers, such as campaigning for clean air programs, enforcing smoke-free legislation, and improving housing conditions, all contribute to a reduction in asthma triggers. School policies that promote asthma-friendly environments, medication access, and staff training ensure fair treatment. Engaging communities and lobbying for supporting legislation fosters an environment in which children with asthma can thrive (79).

## 6 Treatment interventions

**Figure 2:**
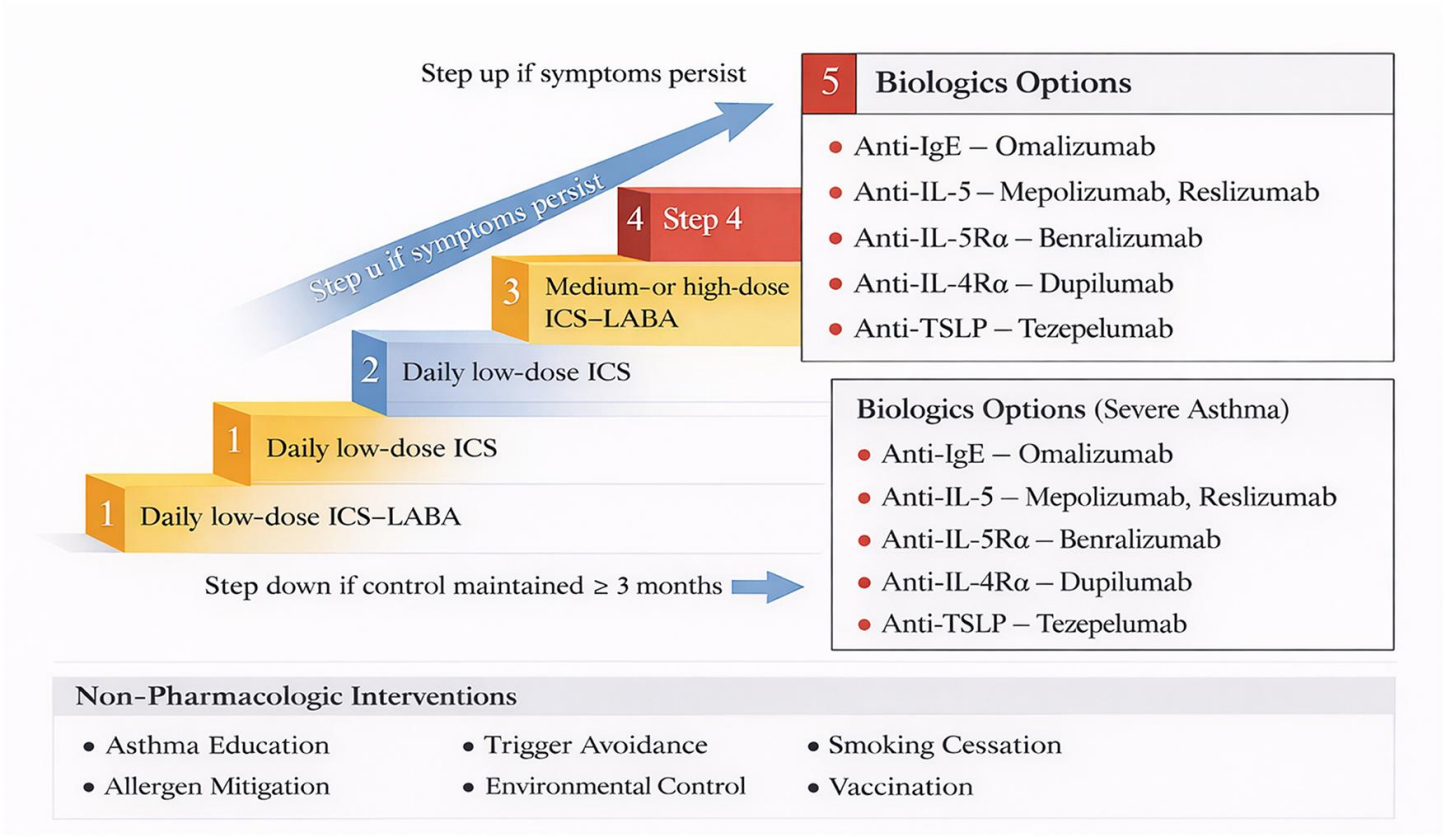
A stepwise strategy to treating pediatric asthma that incorporates biologic agents, pharmaceuticals, and non-pharmacological treatments. The staircase model illustrates how treatment progresses from low-dose ICS-LABA regimens to higher-dose ICS-LABA regimens. When symptoms of severe asthma continue, biologic therapy is added at Step 5. If control is inadequate, treatment is increased; if control is maintained for at least three months, treatment is decreased. At every stage, non-pharmacological treatments are recommended.

The concept of “stepwise therapy”, which modifies drug dosage according to lung function, exacerbation risk, and symptom control, serves as the foundation for treating pediatric asthma. The objective is to minimize side effects while achieving and maintaining control with the least amount of medication that is effective (80). As seen in Figure 2, treatment approaches include pharmacological medicines, biologic therapies, and non-pharmacological measures.

### 6.1 Pharmacological interventions

Controller drugs (long-term prevention) and rescue medications (rapid symptom relief) are two types of pharmaceutical therapies that each have a different function in managing asthma. Children with mild, moderate, or severe chronic asthma must take controller drugs daily to enhance lung function, prevent symptoms, and reduce inflammation (4). For all ages, inhaled corticosteroids (ICS) are the first-line controller because they lower the risk of exacerbation, airway inflammation, and airway hyperreactivity (81). Examples include fluticasone, budesonide, and beclomethasone, with doses weight-based. Side effects are minimal with proper use of oral thrush, and hoarseness can be prevented by rinsing the mouth after use and using a valved holding chamber (82). Long-acting beta-2 agonists (LABA) are used in combination with ICS for moderate to severe persistent asthma (never as monotherapy), providing 12 h bronchodilation. Examples include salmeterol and formoterol, with fixed-dose combinations (e.g., fluticasone/salmeterol, budesonide/formoterol) improving adherence by reducing the number of devices needed (83).

Leukotriene modifiers are oral medications (montelukast, zafirlukast) that block leukotriene-mediated inflammation and bronchoconstriction, useful for children with allergic asthma, exercise-induced bronchoconstriction, or difficulty using inhalers (84). Montelukast (available as chewable tablets or granules) is well-tolerated in preschoolers, making it a flexible option for young children. Inhaled corticosteroid/long-acting muscarinic antagonist (CS/LAMA) combinations are emerging options for children 6 years with moderate to severe asthma (85). LAMAs (e.g., glycopyrronium) provide additional bronchodilation by blocking acetylcholine, and fixed-dose combinations (e.g., fluticasone/glycopyrronium/formoterol) improve control in children with uncontrolled asthma on ICS/LABA (86).

Rescue medications provide rapid relief of acute symptoms and are used as needed. Short-acting beta-2 agonists (SABA) are the first-line rescue medication, relaxing airway smooth muscle within 15 min. Examples include albuterol and levalbuterol, which are administered via a nebulizer, a metered-dose inhaler with a valve and holding chamber, or a dry powder inhaler (24). Overuse of SABA, 2 canisters/year, indicates poor control and the need for controller medication adjustment. Systemic corticosteroids are used for severe exacerbations, reducing severe inflammation but carrying significant side effects with prolonged use. Short courses (3–5days) are safe for acute exacerbations, but long-term use is avoided due to cumulative toxicity (87).

### 6.2 Non-pharmacological interventions

Non-pharmacological interventions complement pharmacological therapy across all stepwise care levels and are built on the daily care and preventive strategies outlined in sections 5.1 and 5.2. Unique non-pharmacological therapy approaches include breathing techniques such as the Buteyko method, diaphragmatic breathing, and pursed-lip breathing, which minimize airway hyperreactivity and enhance symptom control. Age-appropriate training helps children manage symptoms without rescue drugs (88). Complementary lifestyle therapies (e.g., omega-3 supplements, vitamin D, and probiotics (section 5.2.4 for detailed preventive use) may modestly reduce inflammation but are not a substitute for controller medications, and their use should be guided by clinical evidence (89).

**Figure 3:**
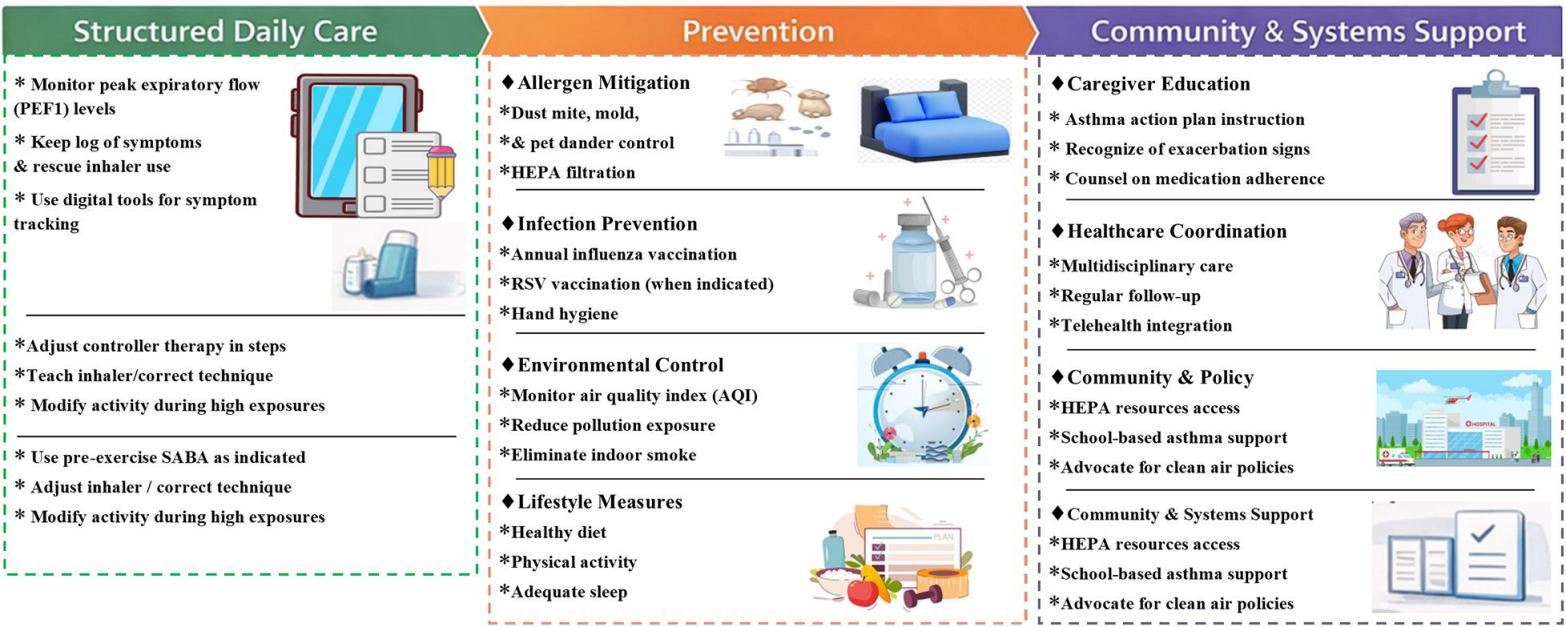
An outline of the preventative programs, structured daily care, and systemic and community-level support measures that work together to help children with asthma. Peak expiratory flow (PEF_1_) monitoring, the use of short-acting β_2_-agonists (SABA), allergen mitigation (e.g., HEPA filtration), infection prevention (e.g., RSV vaccination), environmental control based on air quality index (AQI), and coordinated caregiver, healthcare, and community support are important components.

Stepwise therapy ensures children receive the lowest effective dose by adjusting drug intensity based on clinical symptoms and objective lung function monitoring (e.g., PEF1; section 5.1.1) (Figure 3) (90). All ages use SABA (section 6.1) when necessary for intermittent asthma (step I). Children under the age of six receive montelukast or low-dose ICS for moderate persistent asthma (step 2), whereas those over the age of 2–6 use leukotriene modifiers. Children under the age of six use medium-dose ICS or low-dose ICS + Montelukast for moderate persistent asthma (step 3), whereas children over the age of 2–6 use low-dose LABA or medium-dose ICS. Children under the age of six utilize high-dose ICS montelukast, whereas children over the age of 2–6 require medium or high-dose ICS/LABA LAMA for severe chronic asthma (step 4). For severe uncontrolled asthma (step 5), for all ages, add biologic therapy based on endotype. Treatment should be reviewed every 3–6 months, with step-down if control is maintained for ≥3 months to minimize medication exposure (27, 80, 90). This method ensures that the child receives tailored, adaptable care that adjusts to their changing requirements throughout time.

## 7 Obstacles and gaps in pediatric asthma care

Despite breakthroughs in diagnosis and treatment, pediatric asthma care still faces major obstacles and limitations that influence outcomes, particularly in vulnerable groups. These include challenges to care and adherence, health inequities, and the impact of uncontrolled asthma on families and healthcare systems (21). Addressing these problems is critical to lowering the asthma burden and providing equitable care to all children.

### 7.1 Challenges in accessing care

Many children, particularly those from low-income families, continue to face significant barriers to accessing adequate asthma care. Income households, rural locations, and underserved communities. Challenges include a lack of insurance coverage, restricted access to asthma experts, lengthy appointment wait times, and transportation issues. Children without insurance or with insufficient coverage may delay or refuse critical drugs, diagnostic testing, or emergency care, resulting in uncontrolled symptoms and exacerbations (91). Due to a lack of pediatric asthma experts in rural areas, families must travel great distances for care, which results in logistical and financial difficulties that lower follow-up adherence (92). Medication adherence is a persistent issue in all pediatric age groups, with rates ranging from 30% to 70% for controller drugs. Other impediments include prescription costs, complex regimens needing several daily doses, fear of side effects, and a misunderstanding of the significance of controller drugs. Access to care is also hampered by healthcare system hurdles, such as fragmented treatment with insufficient coordination among primary care providers, specialists, and schools (91). Furthermore, point-of-care diagnostic technologies such as FeNO devices are not generally available in underprivileged clinics, and asthma teaching materials for low-literacy households are limited. Telehealth has emerged as a viable solution for improving access. Especially in rural areas, although gaps in internet access and digital literacy limit its availability to some families (93).

### 7.2 Health inequities and their influence on asthma outcomes

Pediatric asthma health disparities are well documented, with children from racial and ethnic minority groups, low-income households, and urban areas having a higher prevalence, more severe disease, and poorer outcomes. When compared to white children, black, Hispanic, and Indigenous children have higher rates of chronic asthma, frequent exacerbations, emergency department visits, hospitalizations, and asthma-related mortality. These inequities result from a complex interaction of social determinants of health, including poverty, environmental injustice, restricted access to care, and systemic racism (94). Poverty is a major contributor to these discrepancies, since low-income families are more likely to live in substandard housing, which exposes them to indoor allergens such as dust mites, mildew, and cockroaches, as well as tobacco smoke and other pollutants. These families may also have limited access to good food, which contributes to obesity, a recognized risk factor for asthma, and may struggle to afford prescriptions or medical visits. Environmental injustice puts children in low-income and minority neighborhoods at greater risk of exposure to outdoor air pollution from traffic, industrial sites, and waste facilities, which induces inflammation and exacerbates asthma (93). Minority children are less likely than other children to see pediatric asthma experts, receive asthma education (section 5.3.1), or have access to biologic medicines. Systemic racism in healthcare also contributes to mistrust of clinicians, ineffective pain management, and delayed treatment, all of which worsen asthma outcomes. Families with limited English proficiency may struggle to grasp asthma education materials, medication instructions, or asthma action plans, resulting in poor adherence and uncontrolled symptoms (91).

## 8 Future directions

The future of pediatric asthma care depends on improving prevention, diagnostic accuracy, and customized health maintenance while tackling systemic barriers to care (95). New research and interventions shall increasingly emphasize preventive, everyday care routines and health maintenance. New approaches shall prioritize novel diagnostic technologies, environmental and lifestyle techniques, and community-level interventions to reduce asthma burden, with a heavy emphasis on improving daily self-management and prevention. Future breakthroughs in daily asthma treatment will focus on responsible AI and digital health design, addressing justice, ethics, and intersectionality alongside accessibility and engagement (4, 21). Wearable devices (e.g., smart inhalers, breathing-monitoring chest patches) and activity trackers will provide real-time data on medication use, symptoms, and triggers, while AI-powered applications will provide 24 h assistance with symptom assessment and drug troubleshooting (56). To overcome the limitations identified in previous research, future AI development will prioritize diverse, representative training datasets that account for pediatric intersectional identities to prevent perpetuating disparities, as well as strong data privacy safeguards and caregiver-centric permission processes. Furthermore, efforts shall focus on eliminating access hurdles and ensuring AI recommendations are culturally acceptable and accessible to caregivers with different health literacy levels, ensuring technology supports all children, not just privileged subgroups (96, 97).

Prevention research should concentrate on early-life interventions to minimize asthma incidence, particularly in high-risk children. Microbiome-based therapeutics, such as individualized probiotic formulations tailored to a child's gut or airway microbiome, could be investigated to modulate immunological development and minimize allergic sensitization (98). Strategies for early allergen introduction must be enhanced, with individualized guidance based on genetic risk, family history, and microbiome makeup to maximize safety and efficacy. Environmental prevention can be advanced by the development of portable air quality monitors that notify caregivers to excessive pollutant or allergen levels, as well as smart home technologies that modify surroundings in real time to reduce asthma triggers. Vaccines against respiratory viruses can also be developed to lower the major causes of asthma exacerbations, particularly in young children (99, 100).

Addressing the asthma burden at the community level shall necessitate policy changes and collaborative programs that target social determinants of health. Clean air regulations, such as tougher emissions requirements for vehicles and industrial facilities, as well as expenditures in public transportation to reduce traffic pollution, could lower overall community exposure to outdoor contaminants (101). Dwellings' rules shall require mold removal, insect management, and increased ventilation in low-income dwellings to eliminate indoor triggers. School-based preventative programs could be expanded to include comprehensive asthma education for students, faculty, and families, asthma-friendly classroom environments, and integrated physical activity activities to ensure that children with asthma may engage safely. Community health professionals shall play an important role in bridging gaps, delivering in-home asthma education, linking families to services, and lobbying for legislative changes to address local asthma triggers (89).

## 9 Conclusion

Pediatric asthma is a complicated, multifaceted condition that necessitates a comprehensive, tailored approach that prioritizes daily care, health maintenance, and prevention. Symptom monitoring, medication adherence, and a healthy environment are critical for controlling symptoms, reducing exacerbations, and preserving lung function. Targeted protection of allergens, infections, pollution, and lifestyle risks helps to reduce disease burden, while coordination among caregivers, schools, and communities guarantees coordinated treatment. Beyond pharmaceutical therapies, effective management empowers children and families, tackles social and environmental factors, and employs age-appropriate tactics. Despite problems such as access, adherence, and inequities, advances in responsible, equity-centered AI and technology-driven self-management, grounded in addressing intersectionality and ethical considerations, hold promise for reducing disparities in pediatric asthma care. By addressing daily treatment, prevention, and health maintenance, children with asthma can achieve symptom management, flourish in everyday activities, and maintain lifetime lung health, supported by research and policy, and equitable care delivery.
